# Supplementary material for: Enhanced sodium storage in hard carbon via solvent co-intercalation electrolyte enabling Ah-level pouch cells at low temperatures
Source: Nat Commun. 2026 Feb 6;17:1478. doi: 10.1038/s41467-026-69237-y (PMC12886996; doi:10.1038/s41467-026-69237-y)
Supplement: Supplementary file 1 — Supplementary Information [file 41467_2026_69237_MOESM1_ESM.pdf]

Supplementary Information

Enhanced sodium storage in hard carbon via solvent co-intercalation electrolyte enabling Ah-level pouch cells at low temperatures

Meng Li[1], Zeping Liu[1], Yu Zhao[1], Zhaoyu Chen[2], Yu Zhang[3*], Naiqing Zhang[1*]

[1] State Key Laboratory of Urban-rural Water Resource and Environment, School of Chemistry and Chemical Engineering, Harbin Institute of Technology, Harbin, 150001, China

[2] Space Environment Simulation Research Infrastructure, Harbin Institute of Technology, Harbin 150006, China

[3] Department of Chemistry, Stockholm University, Stockholm 10691, Sweden

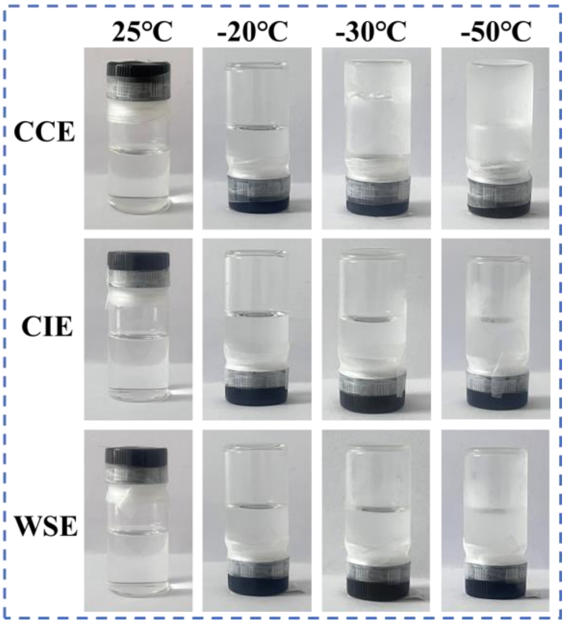

Figure S1. Optical photographs of various electrolytes at different temperatures.

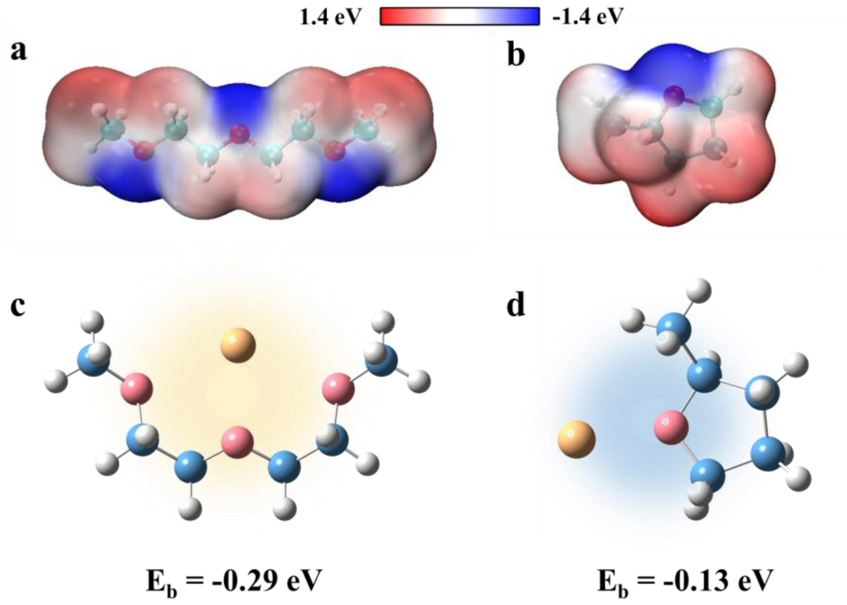

Figure S2. The electrostatic potential and binding energies with Na^+ of (a, c) G2 and (b, d) MO, respectively (yellow spheres represent Na, red spheres represent O, blue spheres represent C, and white spheres represent H).

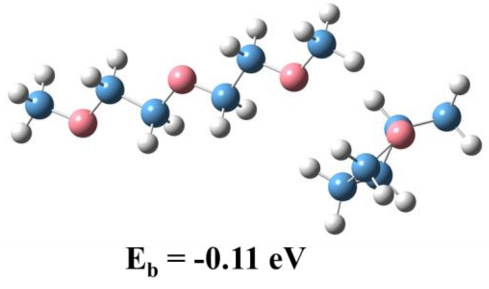

Figure S3. The binding energy between the MO and G2.

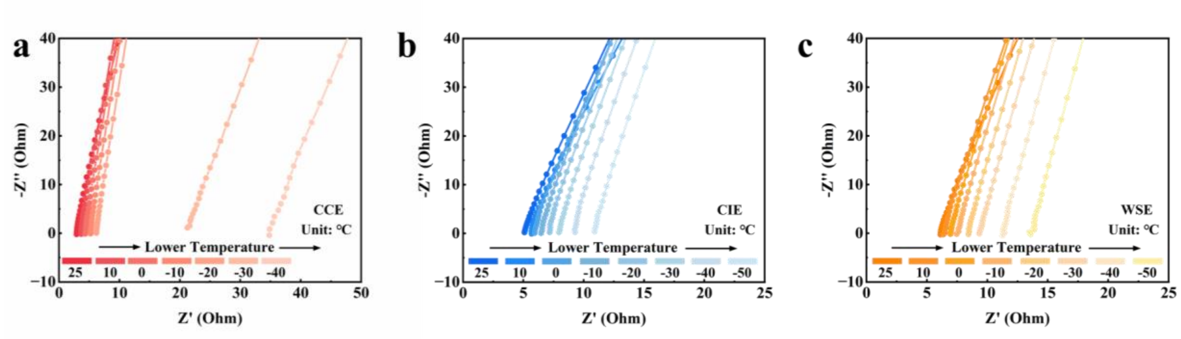

Figure S4. Nyquist plots of (a) CCE, (b) CIE, and (c) WSE at different temperatures.

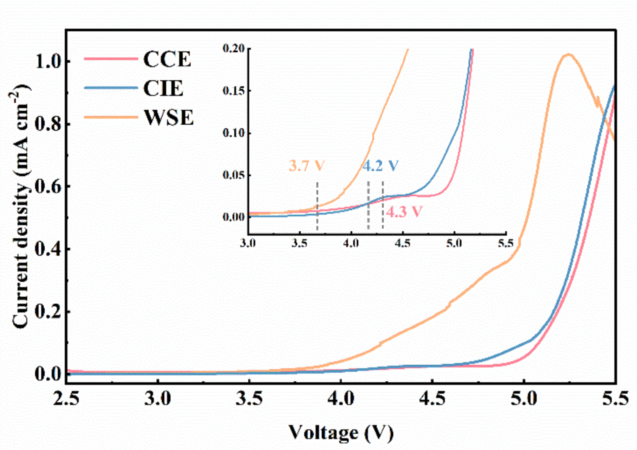

Figure S5. LSV curves of different electrolytes.

We assembled Na||stainless steel cells and evaluated the electrochemical stability window of the electrolyte using linear sweep voltammetry (LSV). The results are shown in Figure S5, where the oxidation decomposition potential for WSE is approximately 3.7 V, CIE is 4.2 V, and CCE is 4.3 V. For SIBs, the typical operating voltage for the positive electrode is around 4 V, and the electrochemical stability window at 4.2 V for CIE ensures that the electrolyte works well within the normal operating voltage range.

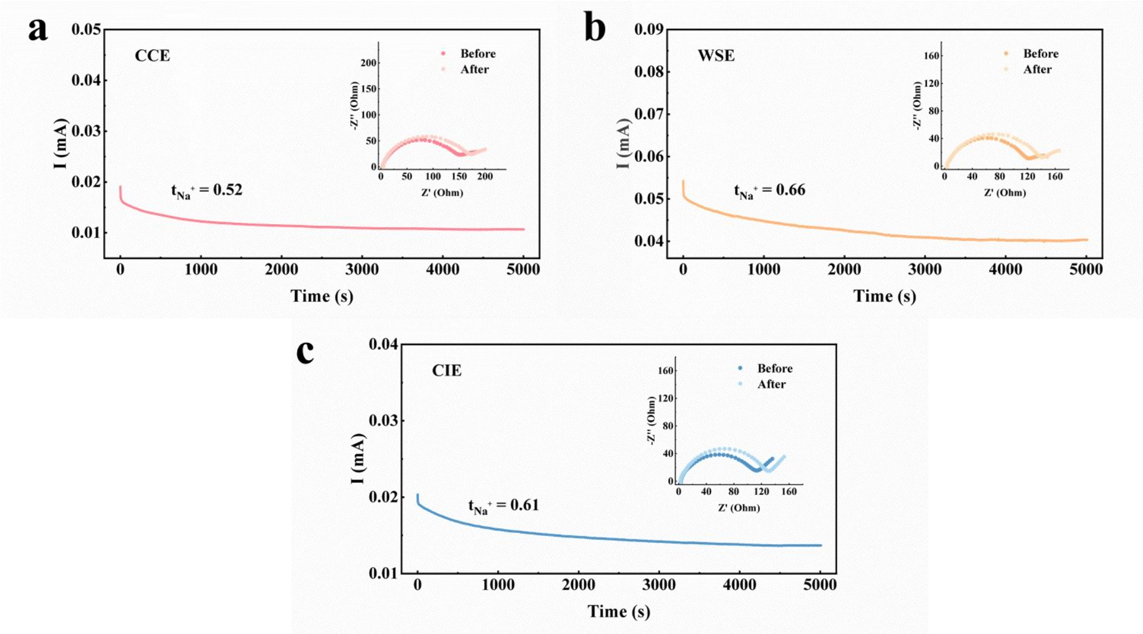

Figure S6. t_+ and corresponding chronoamperometry profiles of Na||Na symmetric cells with (a) CCE, (b) WSE and (c) CIE.

We measured the transference numbers of different electrolytes by combining chronoamperometry curves with AC impedance (Figure S6). Tests were conducted in Na||Na symmetric cells, with transference numbers calculated using the following formula:

$$t_+ = \frac{I_s(\Delta V - I_0 R_0)}{I_0(\Delta V - I_s R_s)}$$

Where I_0 is the initial current, I_s is the steady-state current, R_0 is the pre-polarization impedance, R_s is the post-polarization impedance, and ΔV is the DC bias voltage (10 mV). The calculated t_+ for CCE, CIE and WSE were 0.52, 0.61 and 0.66 respectively. The anion-dominated solvation structure contributes to enhanced ionic transference number, hence WSE has the highest t_+. Due to the introduction of MO, anions are introduced into the outer layer of the solvation sheath, resulting in a higher ion transfer number of CIE than CCE, which is beneficial for reducing polarization and improving rate performance.

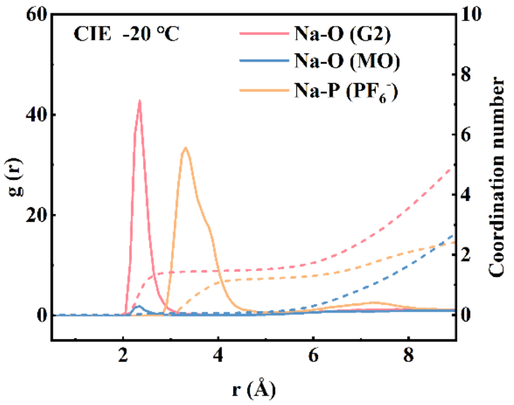

Figure S7. RDF of Na^+ solvation configurations obtained from MD simulations in CIE at -20 °C.

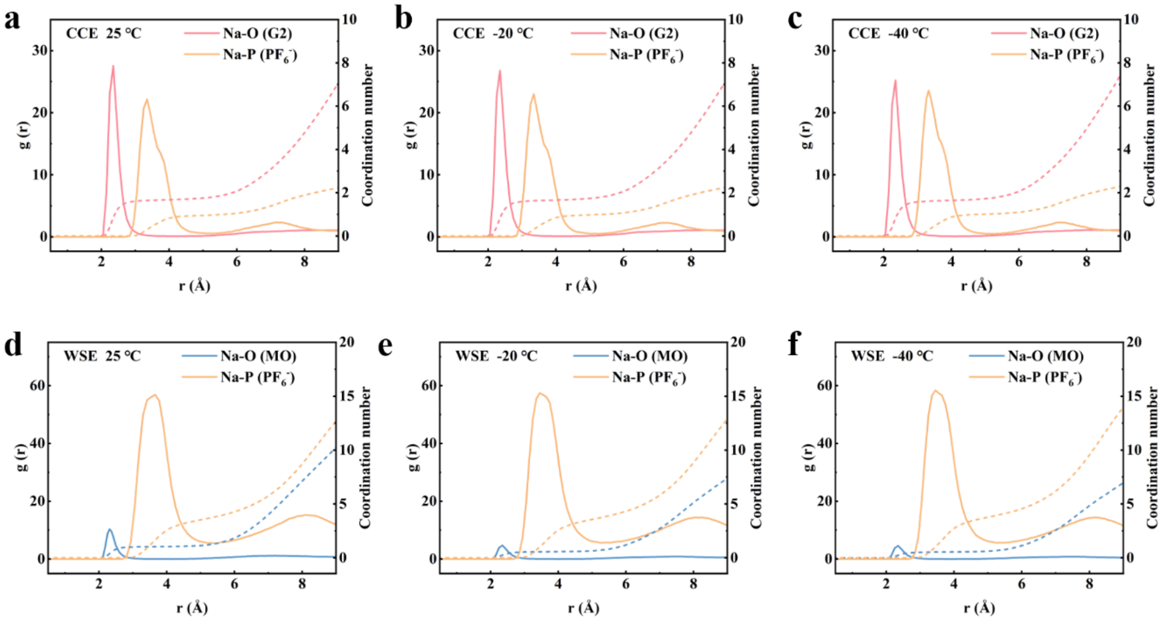

Figure S8. RDF of Na^+ solvation configurations obtained from MD simulations in (a-c) CCE and (d-f) WSE at -40°C - 25°C.

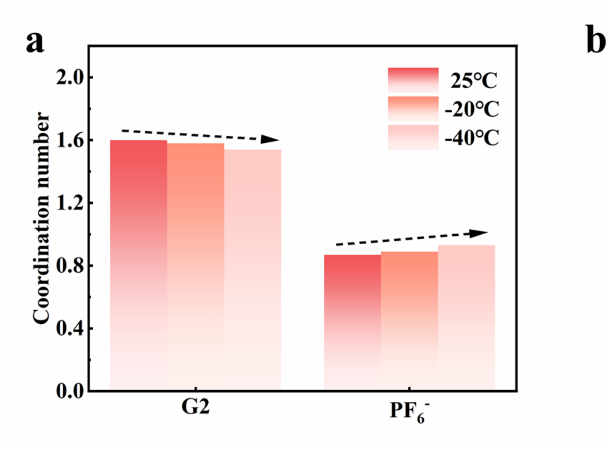

Figure S9. Coordination number changes obtained from MD simulation at different temperatures of (a) CCE and (b) WSE.

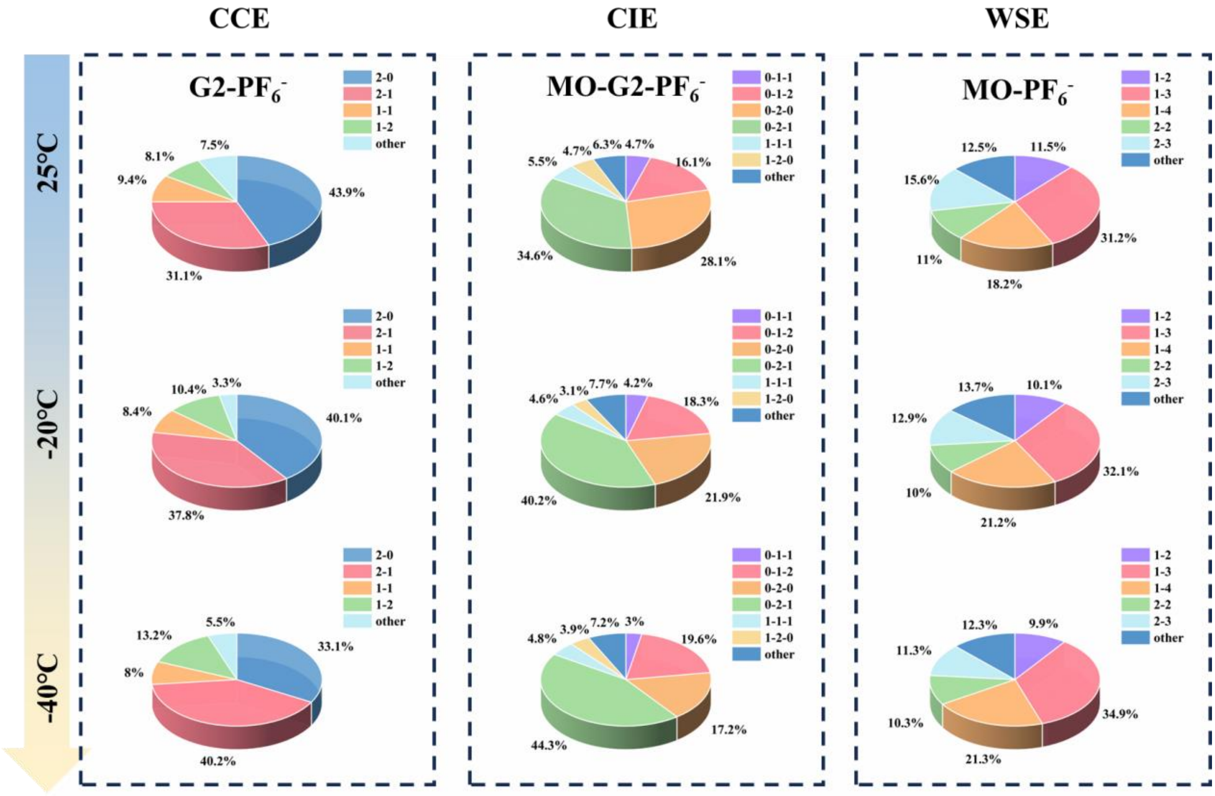

Figure S10. Statistical analysis of solvent-anion coordination environment of various electrolytes at different temperatures.

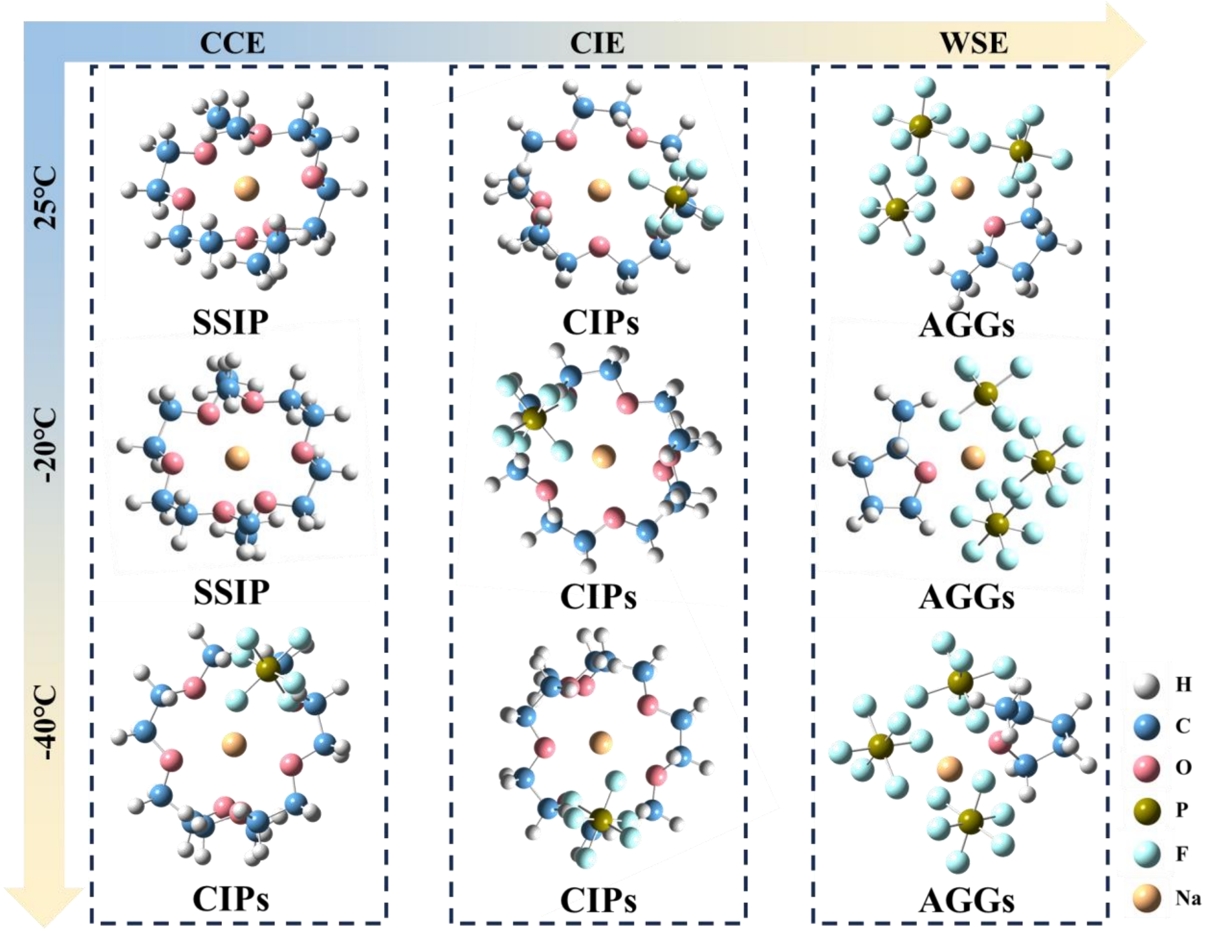

Figure S11. Typical solvation structures of various electrolytes at different temperatures.

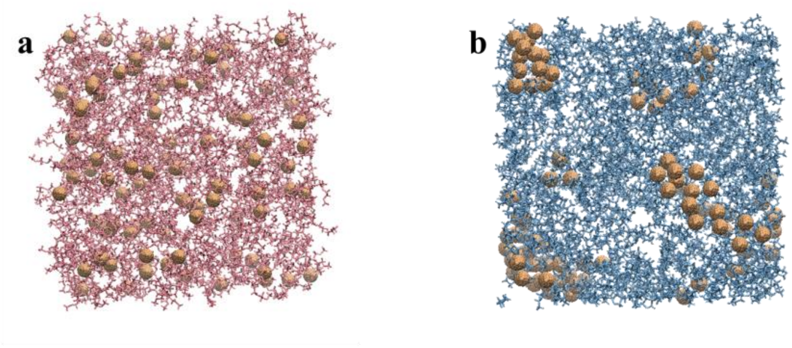

Figure S12. MD simulation snapshot of solvent environment of (a) CCE and (b) WSE, yellow spheres represent sodium, red represents G2 solvent, and blue represents MO solvent.

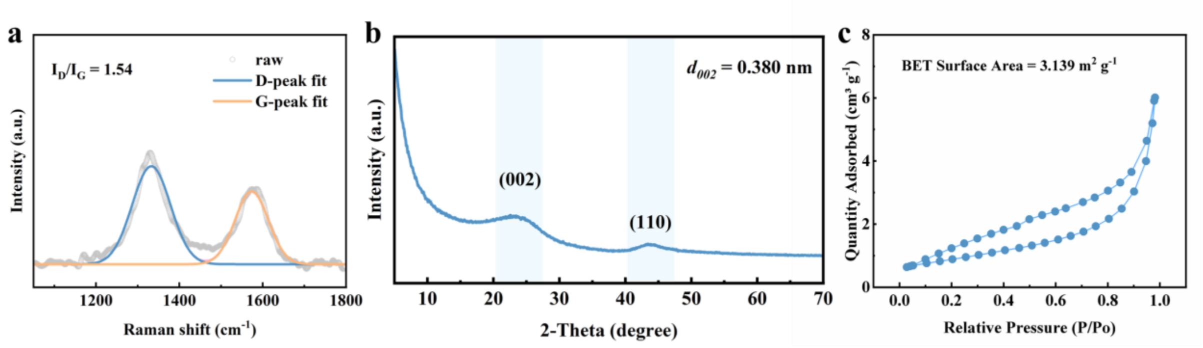

Figure S13. (a) Raman spectra, (b) XRD patterns and (c) N_2 adsorption-desorption isotherms of the hard carbon material used in this work.

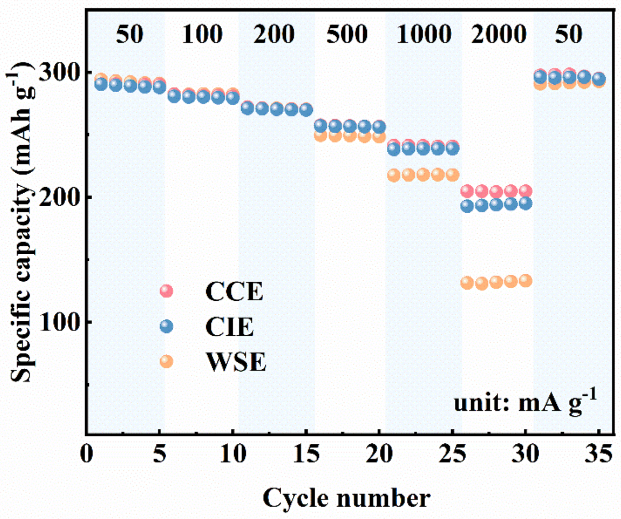

Figure S14. Rate performance of various electrolytes at 25 ℃.

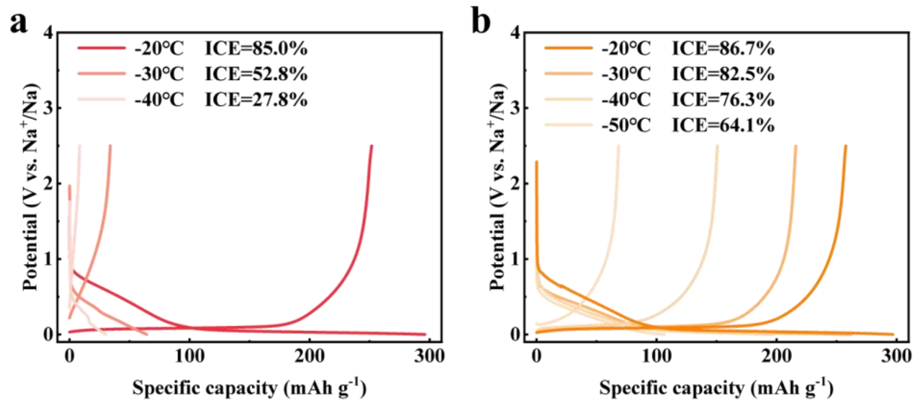

Figure S15. Charge/discharge curves of Na||HC cells at 20 mA g^{-1} in first cycle using (a) CCE and (b) WSE at different temperatures.

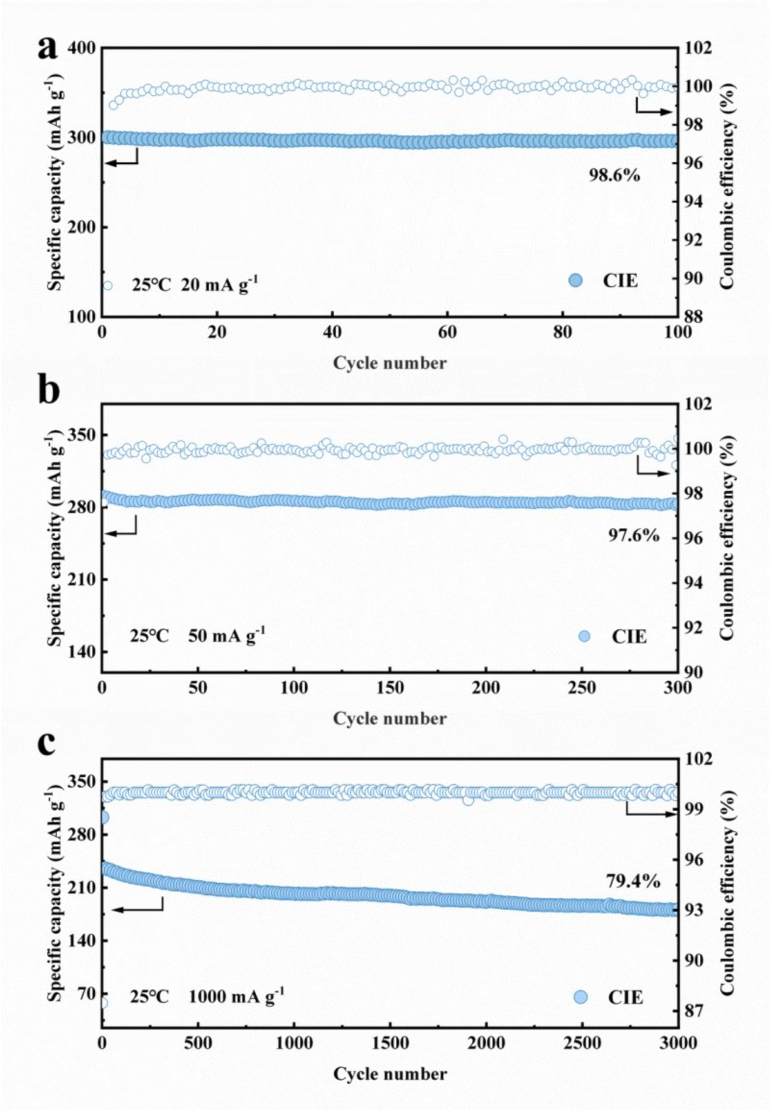

Figure S16. Cycling performance of Na||HC cells using CIE at 25°C with specific current of (a) 20 mA g^{-1}, (b) 50 mA g^{-1} and (c) 1000 mA g^{-1}.

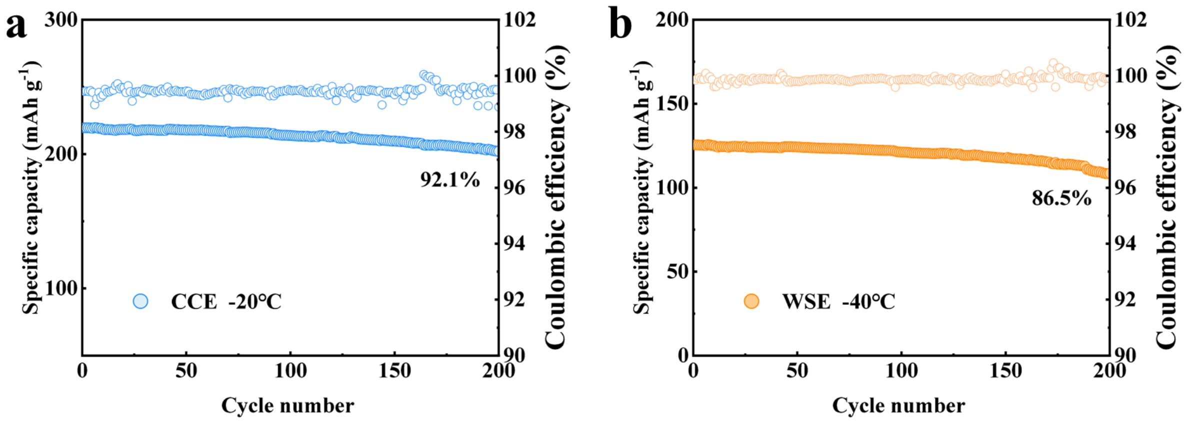

Figure S17. Low temperature cycling performance of Na||HC cells using (a) CCE and (b) WSE at a specific current of 100 mA g^{-1}.

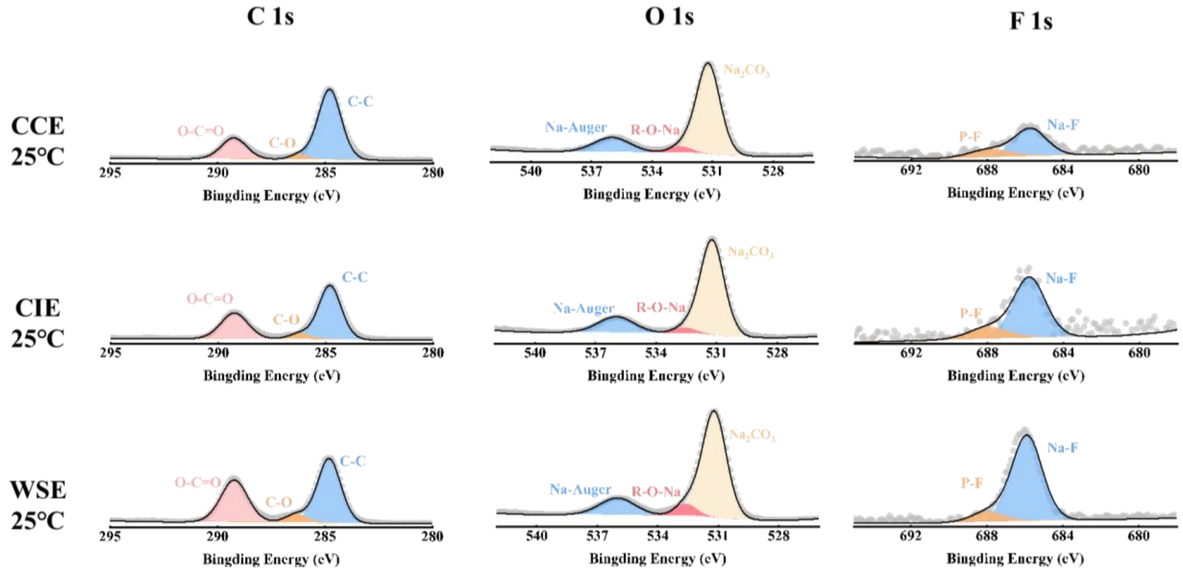

Figure S18. XPS spectra (C 1*s*, O 1*s*, F 1*s*) of HC negative electrodes after 10 cycles (50 mA g^{-1}) using different electrolytes at 25°C.

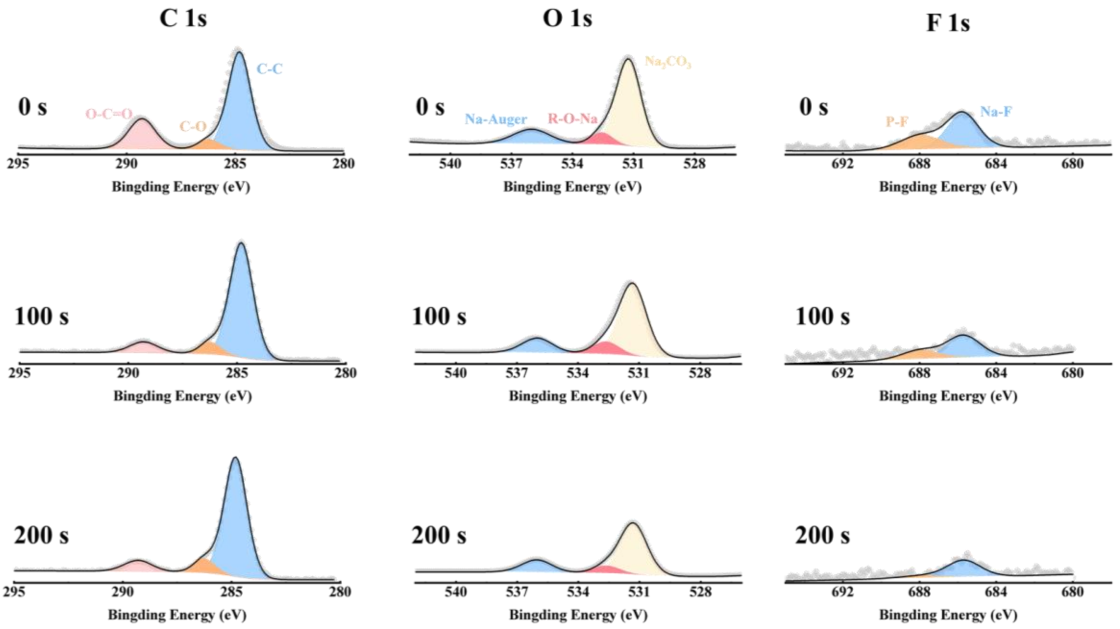

Figure S19. XPS spectra (C 1*s*, O 1*s*, F 1*s*) of HC negative electrodes after 10 cycles (50 mA g^{-1}) at -20°C using CCE with different etching times.

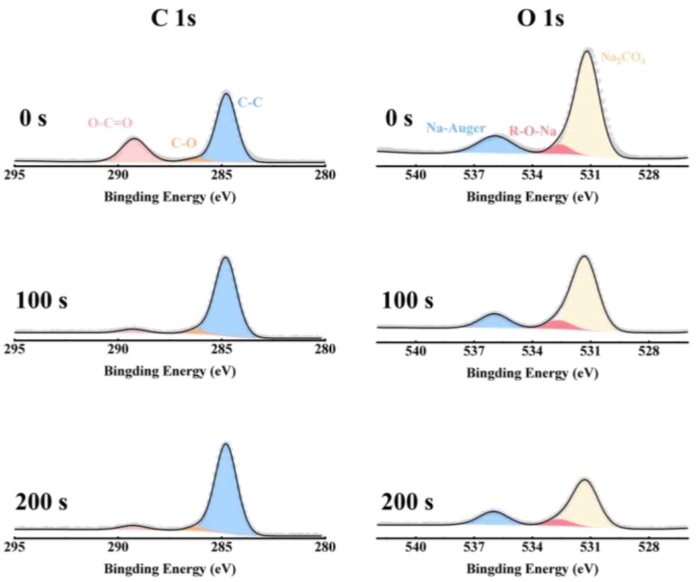

Figure S20. XPS spectra (C 1*s*, O 1*s*) HC negative electrodes after 10 cycles (50 mA g^{-1}) at -40℃ using CIE with different etching times.

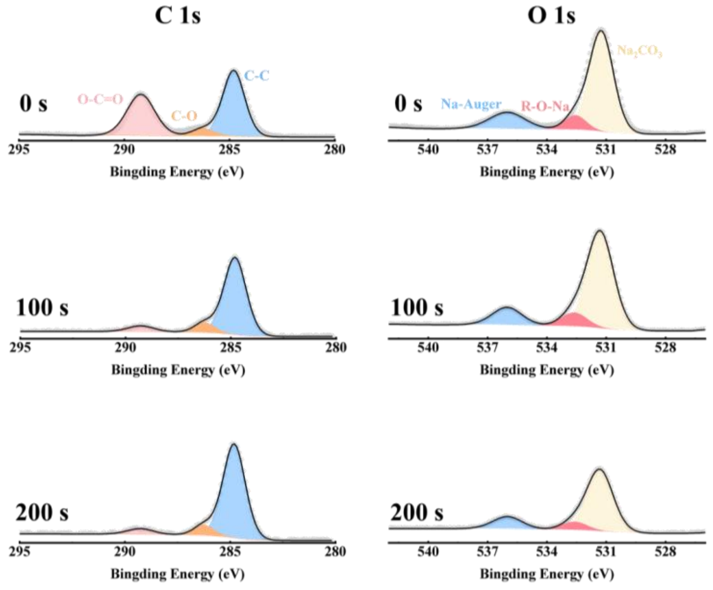

Figure S21. XPS spectra (C 1*s*, O 1*s*) HC negative electrodes after 10 cycles (50 mA g^{-1}) at -40℃ using WSE with different etching times.

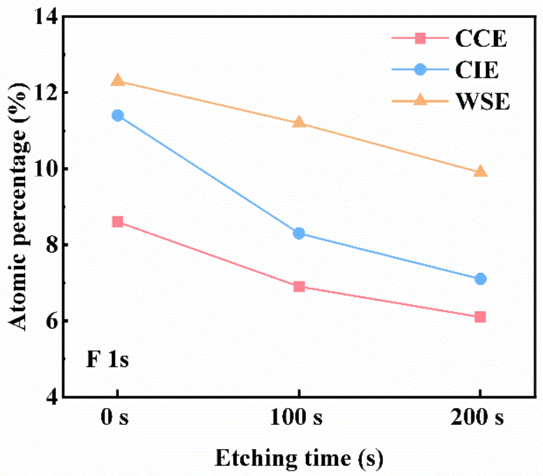

Figure S22. The variation of F element content in SEI layer under different etching times.

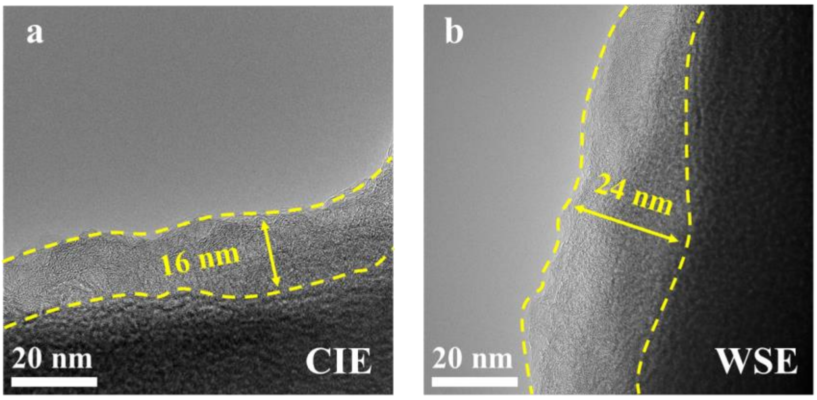

Figure S23. HRTEM images of HC negative electrodes after 10 cycles (50 mA g^{-1}) using (a) CIE and (b) WSE at 25°C.

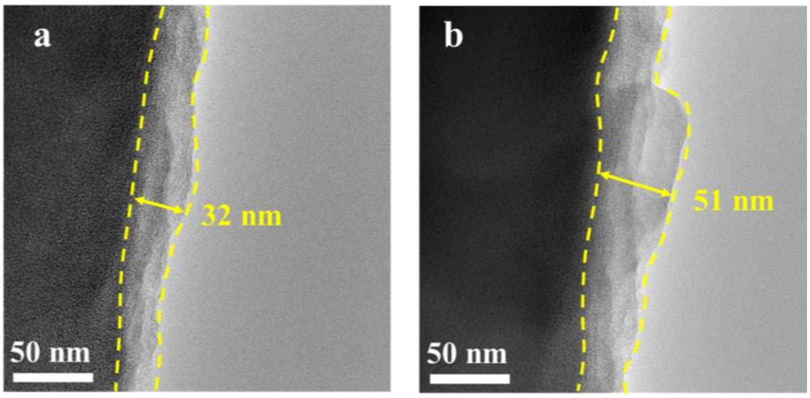

Figure S24. HRTEM images of HC negative electrodes after 10 cycles (50 mA g^{-1}) using CCE at (a) 25°C and (b) -20°C.

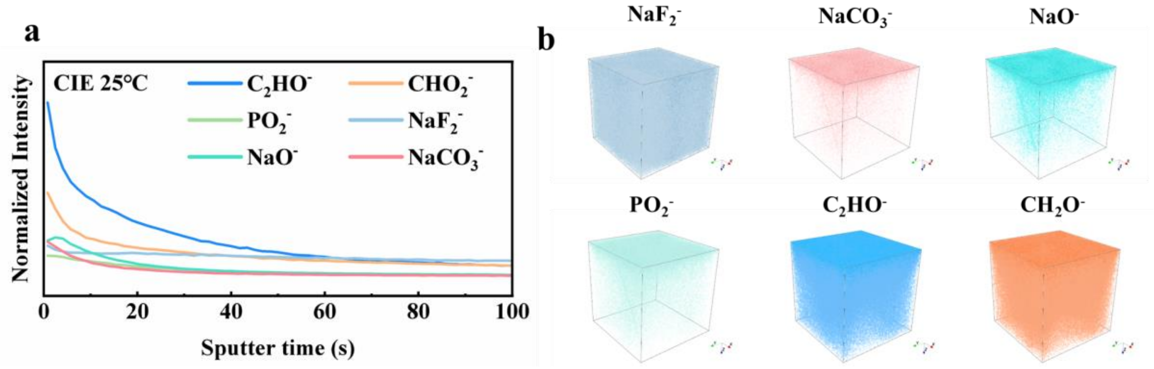

Figure S25. (a) Normalized TOF-SIMS depth profiles of C_2HO^-, CHO_2^-, PO_2^-, NaF_2^-, NaO^- and $NaCO_3^-$ ionic fragments in HC negative electrode after 10 cycles (50 mA g^{-1}) using CIE at 25°C. (b) 3D reconstruction images resolved by TOF-SIMS.

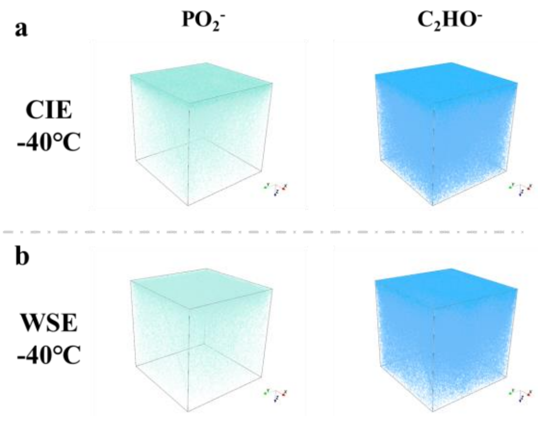

Figure S26. 3D reconstruction images of PO_2^- and C_2HO^- resolved by TOF-SIMS in (a) CIE and (b) WSE at -40°C.

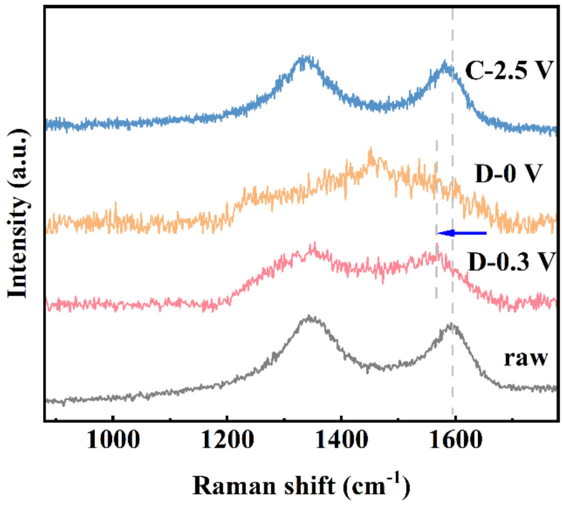

Figure S27. Ex-situ Raman spectra of HC electrodes charged/discharged at a specific current of 50 mA g^{-1} to different potentials using WSE at -40 °C.

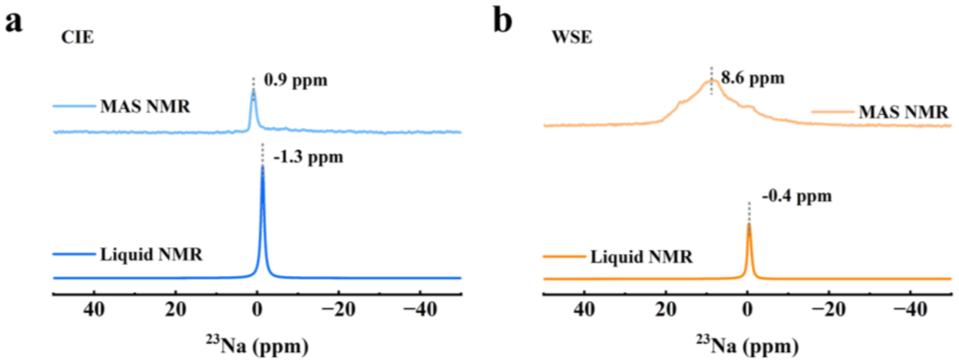

Figure S28. The ^{23}Na NMR spectra of sodiated HC (discharge to 0 V at 50 mA g^{-1}) and electrolytes with (a) CIE and (b) WSE at -40 °C.

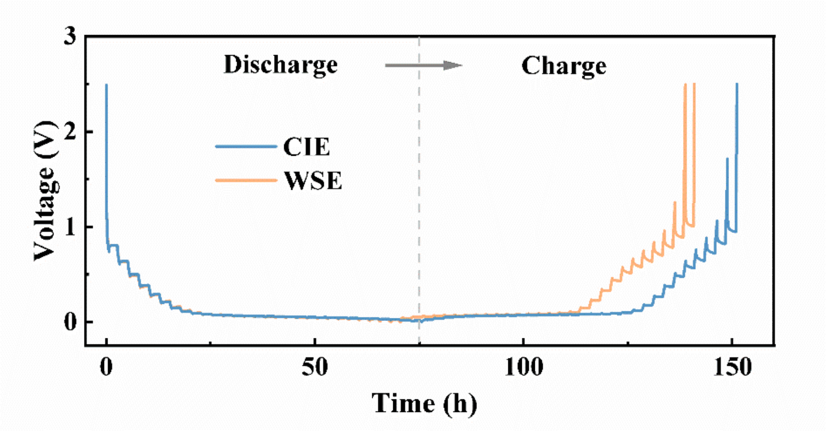

Figure S29. GITT curves of the Na||HC cells at -40 °C.

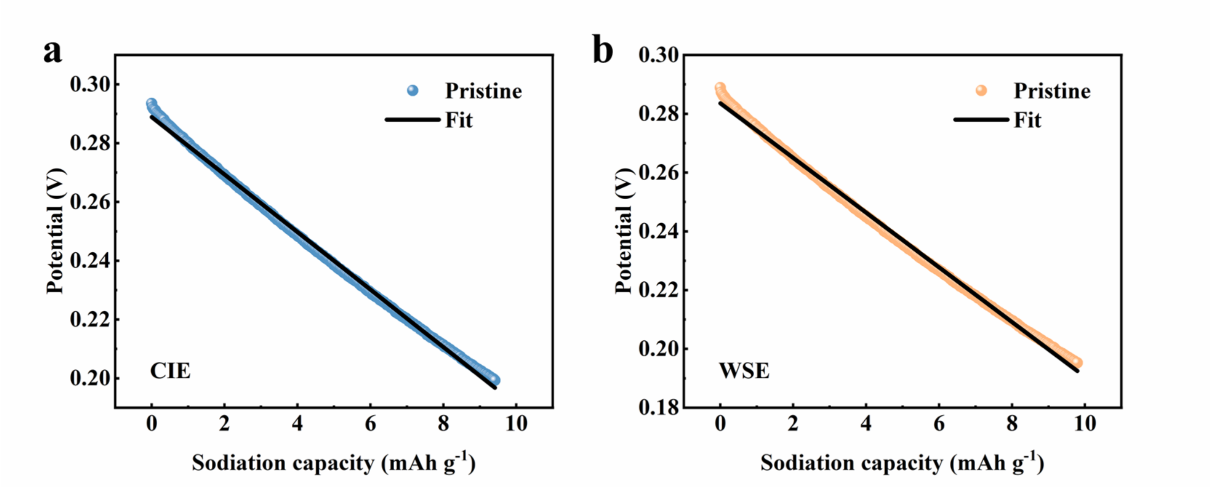

Figure S30. The linear relationship of potential-sodiation capacity during the GITT, (a) CIE, (b) WSE.

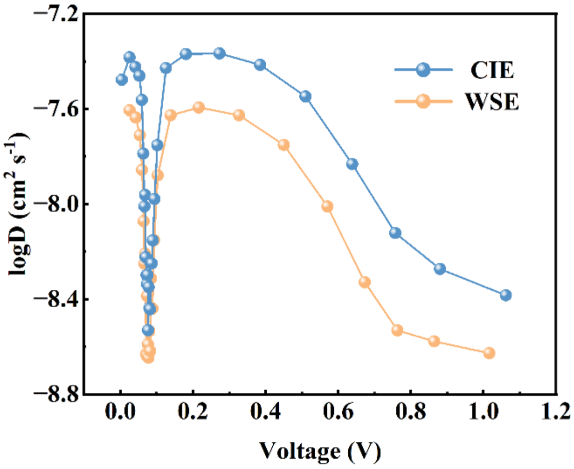

Figure S31. Diffusion coefficients of different electrolytes during the charging process.

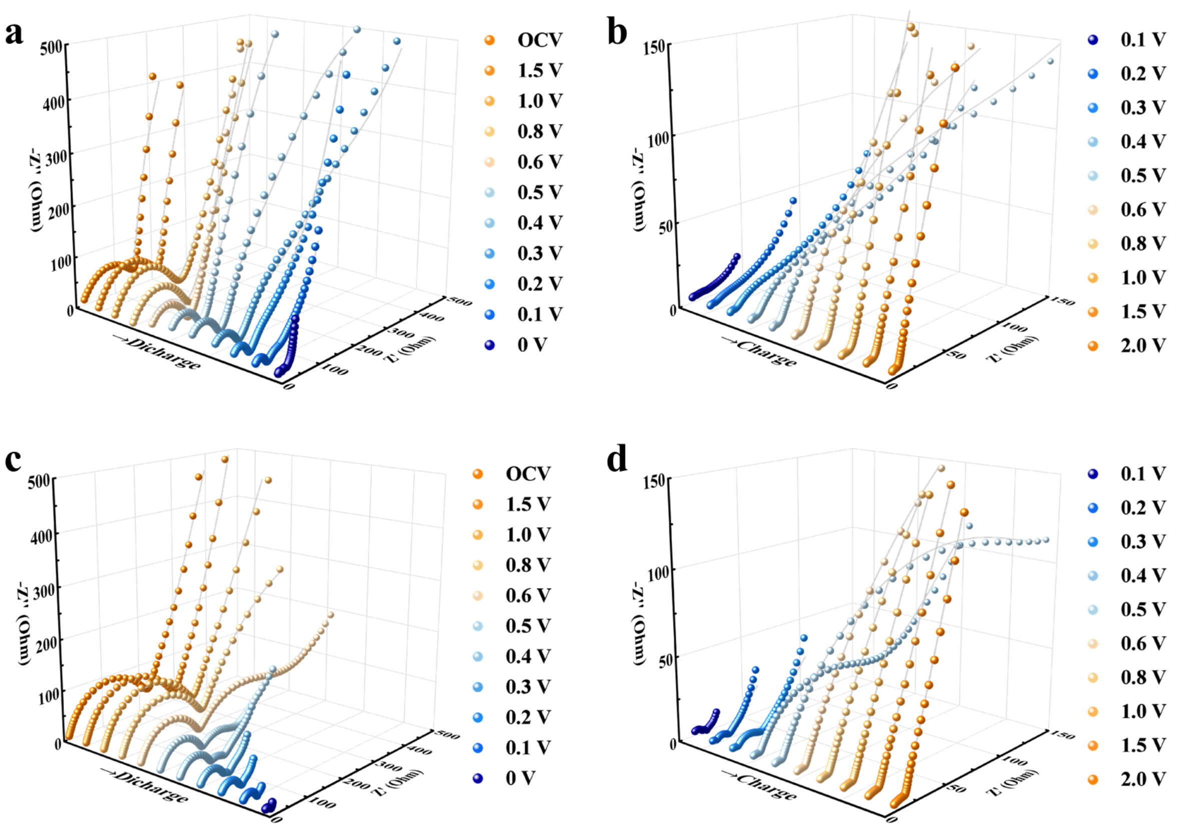

Figure S32. *In-situ* EIS curves during the (a) discharge (50 mA g^{-1}) process of CIE, (b) charge (50 mA g^{-1}) process of CIE and (c) discharge (50 mA g^{-1}) process of WSE, (d) charge (50 mA g^{-1}) process of WSE at -40 °C. (line: fitted data; sphere: pristine data).

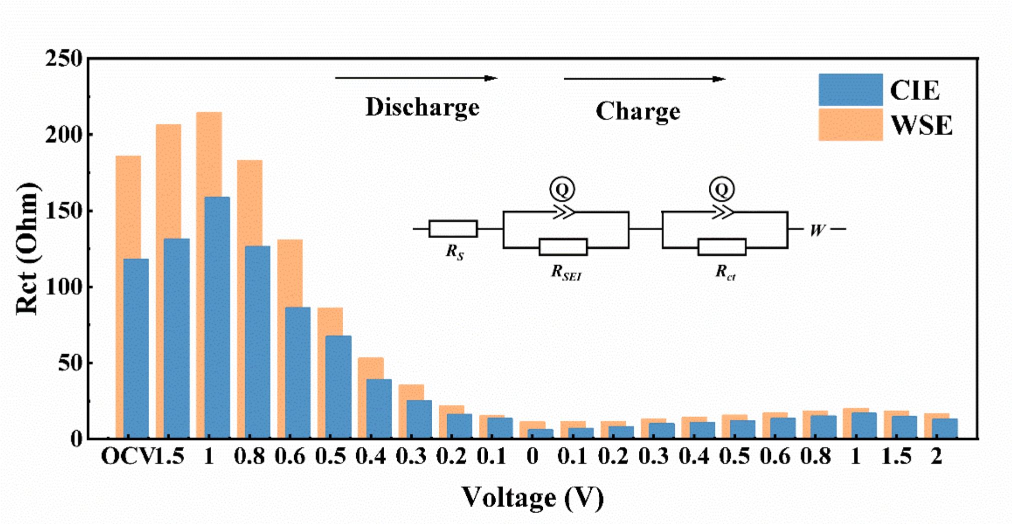

Figure S33. R_{ct} values at different voltages.

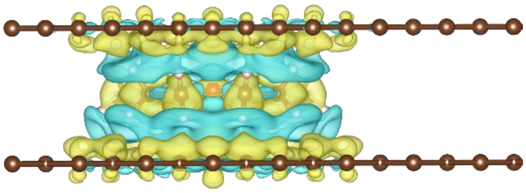

Figure S34. Side views of charge density difference of Na^{+}-G2 in carbon layers.

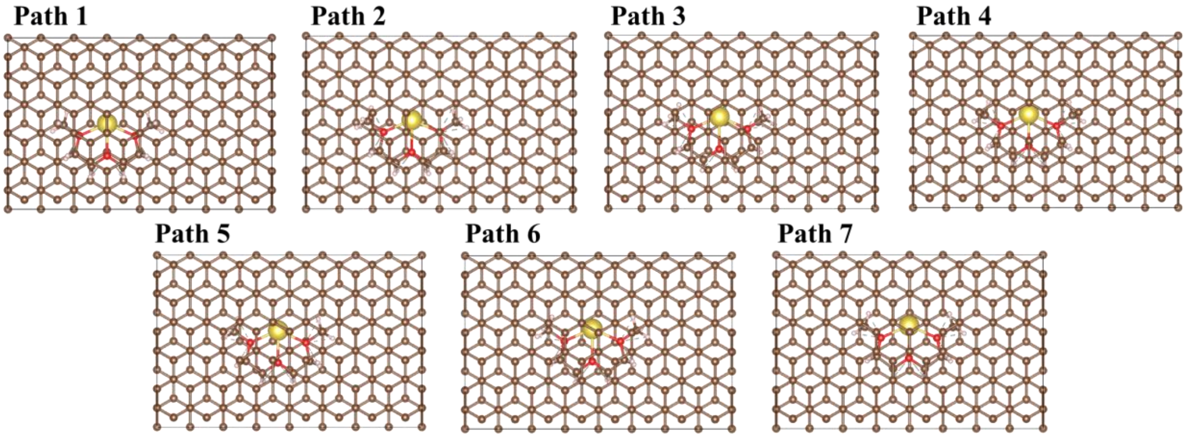

Figure S35. Migration path of Na^{+}-G2 chelate in carbon layer, brown spheres represent C, yellow spheres represent Na, red spheres represent O, and white gases represent H.

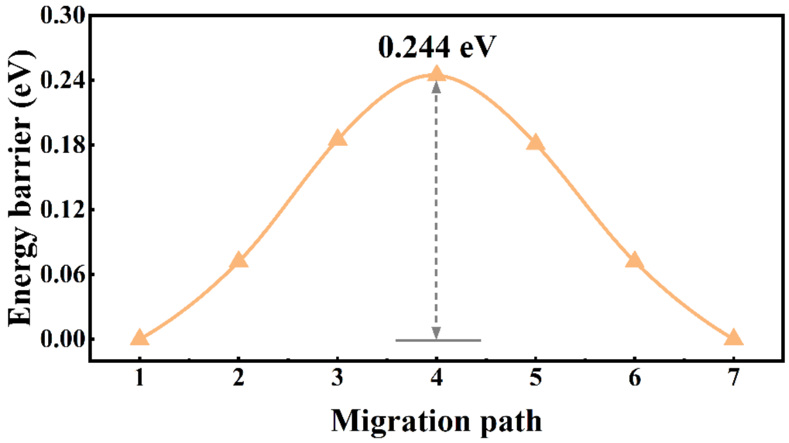

Figure S36. The diffusion energy barrier of a single Na^+.

Table S1. Partial physical properties of solvents.

Solvent	Melting point (°C)	Dielectric constant	Viscosity (mPa s)	Donor number (kcal mol^{-1})
G2	-68	7.2	0.98	19
MO	-136	6.97	0.65	18

Table S2. The specific formulation of different electrolytes.

Electrolyte name	Electrolyte formulation
CCE	0.8 M $NaPF_6$ in G2 = 100 vol%
CIE	0.8 M $NaPF_6$ in G2:MO = 7:3 vol%
WSE	0.8 M $NaPF_6$ in MO = 100 vol%

Table S3. The desolvation energy of different solvents.

	Reaction	ΔG (kJ/mol)
Na^+-$(G2)_n$ (n = 1, 2)	Na^+-$(G2)_2$ →Na^+-$(G2)_1$ + G2	25.5
	Na^+-$(G2)_1$ →Na^+ + G2	28.3
Na^+-MO	Na^+-(MO) → Na^+ + MO	12.3

Table S4. Comparison of ICE of HC at different temperatures between CIE and previously reported SIB electrolytes.

Label	Electrolyte	Room temperature ICE (%)	Low temperature ICE (%)	References
A	$NaPF_6$ in DEGDME + 0.25 M MTPPB	96.6	90.4 (-20°C)	*Angew. Chem. Int. Ed*. 2025, 64, e202416939
B	NaFSI in DMC/TFP	81	/	*Nature Energy* 7, 718–725 (2022)
C	$NaClO_4$ in TMP/TFEP	82.3	/	*J. Am. Chem. Soc.*2024, 146, 23, 15751–15760
D	$NaPF_6$ in PC/TFEP/DEC	82.4	/	*Angew. Chem. Int. Ed.* 2024, 63, e202407717
E	NaTFSI in EC/PC/EP-NaDFOB/SN	70.2	/	*Angew. Chem. Int. Ed.* 2024, 63, e202401051
F	$NaPF_6$ in DME	82.5	60.7 (-20°C)	*Nano Energy* 132 (2024) 110362
G	NaFSI in PC/EC	85	/	*Energy Storage Materials* 16 (2019) 146–154
H	$NaPF_6$ in /EC/DEC	91	/	*Nature Commun* 16, 3634 (2025)
I	NaFSI in AN+ 0.05 M NaDFOB	≈81	/	*Adv. Energy Mater.* 2025, 2405319
J	NaFSI in EC/PC/HFE/FEC	79.94	/	*Nano Energy* 136 (2025) 110777
This work	CIE	93.7	91.4 (-20°C)<br>80.5 (-50°C)	This work

Table S5. Fitting errors of R_{ct} values in the *in-situ* EIS.

	Discharge process										
	OCV	1.5 V	1.0 V	0.8 V	0.6 V	0.5 V	0.4 V	0.3 V	0.2 V	0.1 V	0 V
CIE	1.24%	2.85%	2.88%	7.19%	3.87%	6.29%	1.97%	1.63%	2.28%	4.75%	5.34%
WSE	0.95%	0.78%	1.73%	1.53%	2.03%	2.18%	4.92%	3.20%	5.84%	6.04%	5.59%
	Charge process										
	0.1 V	0.2 V	0.3 V	0.4 V	0.5 V	0.6 V	0.8 V	1.0 V	1.5 V	2.0 V	
CIE	2.89%	3.19%	2.77%	2.81%	3.49%	2.65%	3.96%	5.62%	6.86%	6.55%	
WSE	1.25%	1.08%	0.92%	1.25%	1.13%	2.26%	2.60%	4.87%	5.31%	5.75%	

Table S6. Comparison of the capacity of sodium-ion pouch cells at low temperatures between this work and previously reported.

Label	Capacity (mAh)	Temperature (%)	References
Ref. 52	890.35	-20	*Energy Storage Materials* 73 (2024) 103805
Ref. 26	350	-20	*J. Am. Chem. Soc.* 2025, 147, 5162−5171
Ref. 53	70	-20	*Angew. Chem. Int. Ed.* 2025, 64, e202424028
Ref. 54	1060	-25	*Adv. Mater.* 2024, 36, 2312161
Ref. 55	450	-25	*Adv. Mater.* 2024, 36, 2408161
Ref. 56	729	-30	*Energy Storage Materials* 75 (2025) 103997
Ref. 57	884	-40	*Adv. Energy Mater.* 2025, 15, 2403306
Ref. 58	255	-40	*Angew. Chem. Int. Ed.* 2025, e202502693
This work	1042<br>977<br>790	-30<br>-40<br>-50	This work

Table S7. Pouch cell details and parameters.

	Specifications	Values	Units	Mass (g)
**Positive electrode** (O3-NFM- Super P-CNT- PVDF@Al foil)	Active material ratio	95.3%	/	11.52
	Electrode mass load	15 × 2	mg cm^{-2}	
	Number of layers	8	/	
	Electrode size	60 × 80	mm	
	Reversible capacity	108	mAh g^{-1}	
**Negative electrode** (HC-Super P- CMC-SBR- water@Al foil)	Active material ratio	94.5	/	5.04
	Electrode mass load	6.2 × 2	mg cm^{-2}	
	Number of layers	8		
	Electrode size	62 × 82	/	
	Reversible capacity	300	mAh g^{-1}	
**Separator** (PP)	Number of layers	8	/	0.48
**Current collector** (Al foil)	Mass area density	2.49	mg cm^{-2}	2.01
	Thickness	9	μm	
**Electrolyte** (CIE)	Volume	4	mL	3.80
	Electrolyte-to-capacity (E/C)	3.17	g Ah^{-1}	
**Package**	Al-plastic film and tab	/	/	3.04
**Pouch cell**	Capacity	1.2	Ah	22.85 (without package) 25.89 (with package)
	Average voltage	3.1	V	
	Energy	3.72	Wh	
	Specific energy (without package)	163	Wh kg^{-1}	
	Specific energy (with package)	144	Wh kg^{-1}	

Note: The specific energy reported in Wh kg^{-1} in the manuscript do not consider the packages, we give more detailed cell parameters in the table above and include the packages in the calculations.
